# Environmental enrichment during the chronic phase after experimental stroke promotes functional recovery without synergistic effects of EphA4 targeted therapy

**DOI:** 10.1093/hmg/ddz288

**Published:** 2019-12-09

**Authors:** Antina de Boer, Annet Storm, Maricel Gomez-Soler, Silke Smolders, Laura Rué, Lindsay Poppe, Elena B Pasquale, Wim Robberecht, Robin Lemmens

**Affiliations:** 1 Department of Neurosciences, Experimental Neurology, and Leuven Brain Institute (LBI), KU Leuven—University of Leuven, Leuven 3000, Belgium; 2 VIB, Center for Brain & Disease Research, Laboratory of Neurobiology, Leuven 3000, Belgium; 3 Cancer Center, Sanford Burnham Prebys Medical Discovery Institute, La Jolla, CA 92037, USA; 4 Department of Neurology, University Hospitals Leuven, Leuven 3000, Belgium

## Abstract

Worldwide, stroke is the main cause of long-term adult disability. After the initial insult, most patients undergo a subacute period with intense plasticity and rapid functional improvements. This period is followed by a chronic phase where recovery reaches a plateau that is only partially modifiable by rehabilitation. After experimental stroke, various subacute rehabilitation paradigms improve recovery. However, in order to reach the best possible outcome, a combination of plasticity-promoting strategies and rehabilitation might be necessary. EphA4 is a negative axonal guidance regulator during development. After experimental stroke, reduced EphA4 levels improve functional outcome with similar beneficial effects upon the inhibition of EphA4 downstream targets. In this study, we assessed the effectiveness of a basic enriched environment in the chronic phase after photothrombotic stroke in mice as well as the therapeutic potential of EphA4 targeted therapy followed by rehabilitation. Our findings show that environmental enrichment in the chronic phase improves functional outcome up to 2 months post-stroke. Although EphA4 levels increase after experimental stroke, subacute EphA4 inhibition followed by environmental enrichment does not further increase recovery. In conclusion, we show that environmental enrichment during the chronic phase of stroke improves functional outcome in mice with no synergistic effects of the used EphA4 targeted therapy.

## Introduction 

Worldwide, stroke is the main cause of long-term adult disability (1). Although mortality rates are decreasing, the global burden of stroke is increasing. Both the aging population and the high numbers of chronically disabled stroke survivors contribute to this high global burden (1,2). Therefore, therapies enhancing post-stroke recovery are of interest. Stroke pathology and recovery involve three specific phases. The acute phase, covering the first hours to days after stroke, is characterized by rapid cell death and inflammation. After the first week to about 3 months post-stroke, endogenous recovery mechanisms result in rapid functional improvements, the subacute phase. From 3 months on, patients enter a chronic phase in which functional recovery reaches a plateau that is partly modifiable by intense rehabilitation (3–5). The extent of recovery varies among stroke patients and strongly depends on lesion type, lesion size and the severity of the initial deficit (6,7).

Similar to human stroke, *in vivo* stroke models show rapid subacute recovery and plasticity within the first week, with additional improvements in later stages if rehabilitative training is applied (8). A variety of rehabilitation paradigms can be used after experimental stroke, including skilled reaching tasks and enriched environments (9,10). Previous studies identified a time window of effective rehabilitation. Hyperacute rehabilitative training possibly worsens the initial deficit while subacute rehabilitation improves behavioral outcome with efficacy of rehabilitation declining with time (11–13). Underlying mechanisms are likely similar to those seen during subacute spontaneous recovery, i.e. altered expression of axonal growth-promoting and -inhibitory genes, changes in astrocyte reactivity and glial scar formation and structural remapping in the motor cortex, subcortical areas and corticospinal tract (CST) pathways (14).

After experimental stroke, subacute activation of growth-promoting factors encourages sprouting of axons, dendrites and spines needed for axonal rewiring (15). Subsequent return to a growth-inhibitory environment counterbalances this response to limit aberrant neurite outgrowth or repel sprouting axons (16). Many different growth-inhibitory molecules are present including myelin structures, glial scar components and several developmental axonal guidance cues like EphA4 (17). EphA4 is a member of the Eph system, a large family of receptor tyrosine kinases that serve as important regulators of axonal guidance during development (18). EphA4 interacts with ephrin ligands causing bi-directional signaling resulting in effects in the cell expressing the receptor as well as the cell bearing the ligand (19). In general, EphA4 downstream signaling causes actin cytoskeletal changes leading to growth cone collapse which limits axonal outgrowth (20).

Several studies show that blocking axonal growth-inhibitory molecules stimulates axonal plasticity and improves stroke recovery (21,22). Additionally, combining rehabilitation with such a therapy might serve as the optimal strategy to maximize post-stroke functional improvement as was shown by treating rats with anti-Nogo-A antibodies for 2 weeks post-stroke followed by intense rehabilitative training (23,24). Previously, we showed that constitutive EphA4 knockdown improves stroke outcome, and blocking EphA4 downstream signaling results in a similar beneficial effect (25). Furthermore, EphA4 is upregulated in post-stroke sprouting neurons in aged compared to younger rats (26), possibly contributing to reduced recovery potential in aged animals. Subacute Eph-ephrin inhibition results in structural remapping of ipsilesional cortical areas and improves functional recovery (27). These findings suggest that inhibition of EphA4 combined with rehabilitative training might serve as a novel therapeutic strategy to enhance functional recovery after stroke.

In this study, we assessed the effect of subacute EphA4 targeted therapy in combination with environmental enrichment during the chronic phase after photothrombotic stroke. We assessed both the efficacy of the enriched environment as well as the possible therapeutic relevance of EphA4 inhibition in combination with environmental enrichment to improve stroke recovery.

## Results 

### EphA4 is expressed in the majority of surviving neurons after experimental stroke

**Figure 1:**
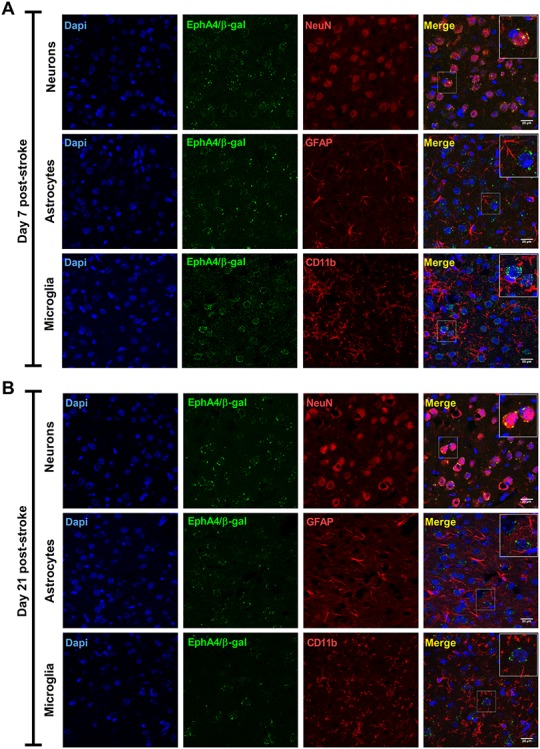
EphA4 is expressed in ipsilesional cortical neurons during the subacute and chronic phase after stroke. (**A**) Immunostaining for the neuronal marker (NeuN), astrocytic marker (GFAP) and microglial marker (CD11b) shows the presence of EphA4/β-galactosidase (EphA4/β-gal) in periinfarct neurons at 1 week after experimental stroke while no protein was detected in astrocytes and microglia. (**B**) At 3 weeks post-stroke, a similar staining pattern was observed with EphA4/β-gal in perilesional neurons and no expression in astrocytes and microglia. EphA4/β-gal = EphA4/β-galactosidase fusion protein, NeuN = neuronal nuclear protein, GFAP = glial fibrillary acidic protein, CD11b = cluster of differentiation 11b. Scale bar = 20 μm.

To study the role of EphA4 targeted therapy in stroke recovery, we first examined EphA4 cell-type specific expression within the motor cortex using an EphA4 reporter mouse (EphA4^LacZ^) (28). In these animals, the endogenous EphA4 promotor is followed by a LacZ gene producing an EphA4/β-galactosidase (EphA4/β-gal) fusion protein. The EphA4/β-gal protein can easily be detected with β-gal antibodies allowing the identification of EphA4 expression in specific cell types or tissues. We used this approach since it is more reliable than immunodetection with anti-EphA4 antibodies. Double immunostainings with antibodies against β-gal and neuronal (NeuN), astrocytic (GFAP) or microglial (CD11b) markers identified the presence of the EphA4/β-gal fusion protein in neurons at 2 weeks (Fig. 1A) and 3 weeks (Fig. 1B) after experimental stroke in EphA4^LacZ^ mice. We detected no EphA4/β-gal fusion protein in astrocytes and microglia at these time points.

**Figure 2:**
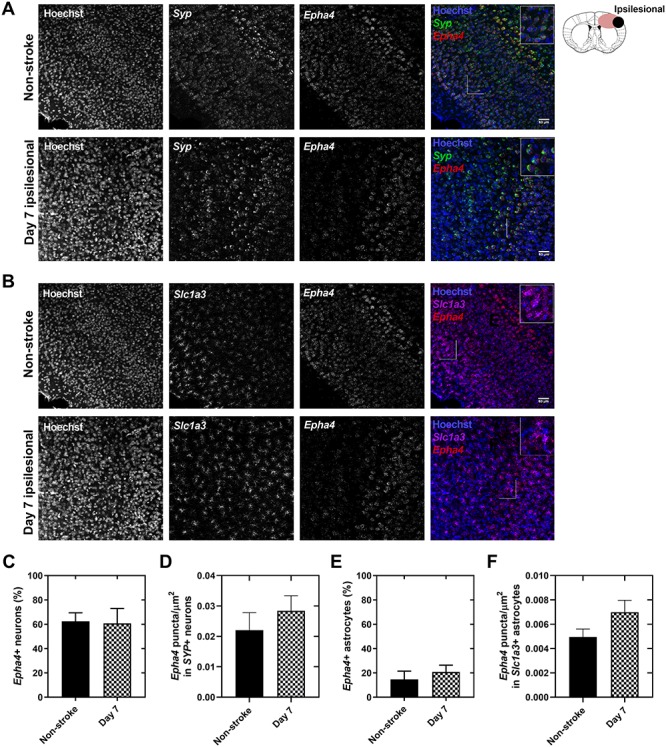
Neuronal and astrocytic *Epha4* mRNA detection at 1 week after experimental stroke using RNAscope *in situ* hybridization. (**A** and **B**) In C57BL/6J (WT) mice, 63% of *Syp+* neurons express *Epha4* at baseline with no change at 1 week post-stroke (61%). (**C**) *Epha4* expression levels identified as the total number of *Epha4* puncta/μm^2^ was unaltered at 1 week after stroke (0.028 puncta/μm^2^) compared to baseline (0.022 puncta/μm^2^) in *Syp +* neurons. (**D** and **E**) 10% of *Slc1a3+* astrocytes expressed *Epha4* under baseline conditions and 15% at 1 week after stroke. (**F**) The average number of *Epha4* puncta/μm^2^ was low in astrocytes (0.005 puncta/μm^2^) at baseline conditions. No differences were observed between stroke (0.007 puncta/μm^2^) and baseline conditions. Data are presented as mean ± SD, Student’s t-test, *n* = 3 mice per condition, *P* > 0.05. *Syp* = synaptophysin, *Slc1a3* = solute carrier family 1 member 3. Scale bar = 50 μm.

Other groups have documented astrocytic *Epha4* gene expression after experimental stroke and other CNS injury models (27,29). We validated the lack of astrocytic EphA4 in EphA4^LacZ^ mice at a single cell level using another method. In C57BL/6J mice, RNAscope *in situ* hybridization with probes specific for neurons (*Syp*), astrocytes (*Slc1a3*) and EphA4 (*Epha4*) showed that at 1 week post-stroke, *Epha4* was expressed in 61% of *Syp* + neurons (Fig. 2A and B) and 15% of *Slc1a3+* astrocytes (Fig 2D and E). The percentage of *Epha4+* neurons and *Epha4+* astrocytes did not change compared to baseline non-stroke conditions (63 and 10%, respectively). In general, astrocytes expressed *Epha4* only at low levels; 0.005 ± 0.001 *Epha4* puncta/μm^2^ compared to 0.022 ± 0.006 *Epha4* puncta/μm^2^ in neurons (Fig. 2C and F). Limited astrocytic *Epha4* expression explains the lack of astrocytic EphA4/β-gal protein detection in EphA4^LacZ^ mice. Comparing stroke and non-stroke conditions, no changes in *Epha4* puncta/μm^2^ in neurons nor astrocytes were detected (Fig. 2C and F).

### EphA4 levels are increased after experimental stroke while EphA4 phosphorylation remains unaltered 

**Figure 3:**
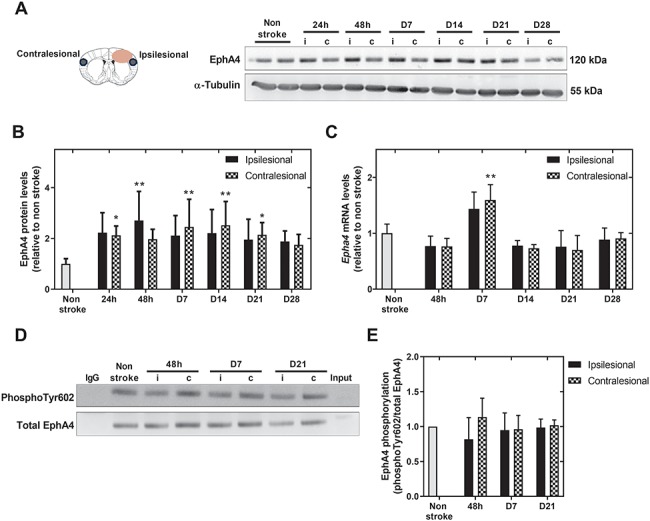
EphA4 temporal expression and activation in the ipsi- and contralesional cortex after experimental stroke. (**A** and **B**) Compared to non-stroke conditions, total EphA4 protein levels were increased from 24 h to 3 weeks after stroke. (**C**) *Epha4* mRNA levels increased at 1 week post PTL and returned to baseline levels from day 14 onwards. (**D** and **E**) EphA4 phosphorylation levels identified as the fraction of phosphotyr602 compared to total EphA4 levels remained stable during the acute (48 h), subacute (day 7) and chronic (day 21) phases after stroke. All data are presented as mean ± SD, one-way ANOVA, *n* = 3–5 mice per time point, ^*^P < 0.05, ^**^P < 0.01 compared to non-stroke. i = ipsilesional, c = contralesional, phosphoTyr602 = phosphorylation of tyrosine residue 602.

To determine EphA4 protein and mRNA levels, cortical brain tissue from C57BL/6J mice was collected from the ipsi- and corresponding contralesional cortex at 24 and 48 h, day 7, 14, 21 and 28 after photohrombotic stroke. EphA4 protein levels increased in the ipsi- and contralesional cortex and returned to baseline levels at 4 weeks after stroke (Fig. 3A and B). Although we did not detect post-stroke differences in *Epha4* puncta/μm^2^ in neurons nor astrocytes using *in situ* hybridization (Fig. 2C and F), total *Epha4* mRNA levels in cortical tissue were elevated at day 7 post-stroke (Fig. 3C). Eph-ephrin signaling depends on Eph intrinsic phosphorylation and conformation changes facilitating activation of the kinase domain. After experimental stroke, EphA4 phosphorylation levels at one of its tyrosine residues (Tyr602) remained stable during the acute (24 h), subacute (day 7) and chronic phase (day 21) (Fig. 3D and E).

### Basic subacute environmental enrichment effectively improves stroke outcome

**Figure 4:**
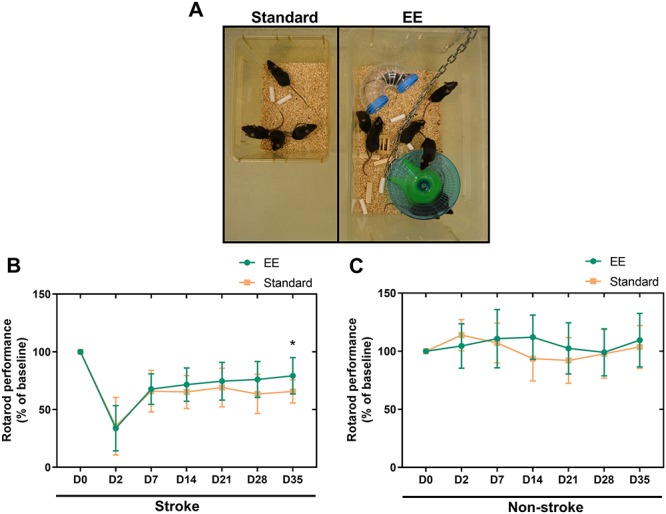
A basic enriched environment improves functional motor performance after experimental stroke without modifying general motivation or skilled movement. (**A**) Environmental enrichment to provide social, sensorimotor and cognitive stimulation. Eight to ten mice were placed in a large cage with multiple rodent toys, including a climbing ladder (not shown), running wheel, different shaped object and tunnels while standard housing consisted of 3–5 animals per mouse cage without additional toys. (**B**) Environmental enrichment improved stroke recovery at 5 weeks after stroke as shown by enhanced rotarod performance in mice exposed to an enriched environment versus animals in standard housing conditions. Two-way ANOVA, *n* = 20–22 mice per group, ^*^P < 0.05. (**C**) Rotarod performance in non-stroke animals remained unaffected over the course of 5 weeks. Two-way ANOVA, *n* = 9–10 mice per group, *P* > 0.05. Data are presented as mean ± SD. EE = enriched environment.

To implement rehabilitation in our functional EphA4 targeted experiment, we first assessed the effectiveness of a basic enriched environment on functional outcome after experimental stroke. C57BL/6J mice were trained on the accelerating rotarod, followed by a baseline measurement prior to photothrombotic stroke. At day 2 post-stroke, mice were functionally tested confirming clear functional deficits compared to baseline performance. After testing, animals were randomly assigned to either enriched or standard housing conditions (Fig. 4A). Spontaneous recovery occurred in mice in standard cages as shown by improved performance on the accelerating rotarod (Fig. 4B). Exposure to an enriched environment ameliorated functional outcome compared to standard housing at 5 weeks after experimental stroke (Fig. 4B). Assigning mice to environmental enrichment did not modify general activity, motivation or skilled movement as rotarod performance over the course of 5 weeks was similar in non-stroke animals in enriched versus standard housing conditions (Fig. 4C).

### EphA4 and ephrin ligand levels remain unaltered upon environmental enrichment

**Figure 5:**
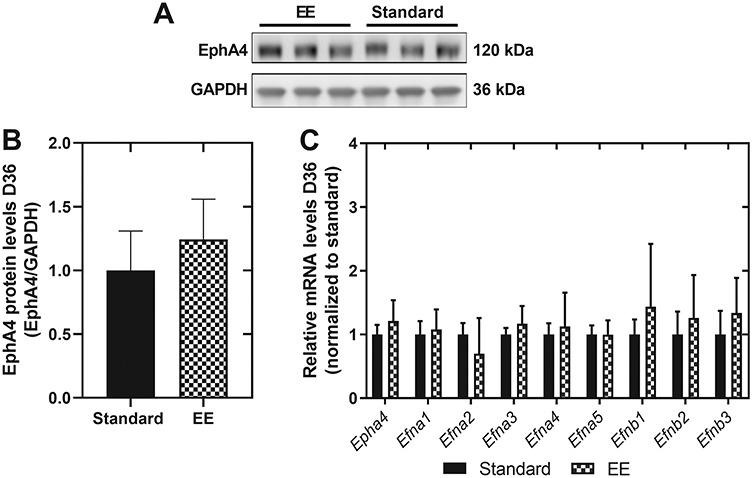
The introduction of an enriched environment does not change EphA4 and ephrin ligand expression after 5 week behavioral follow-up. (**A** and **B**) EphA4 protein levels in ipsilesional cortical brain tissue of mice exposed to enriched or standard housing at day 36 post-stroke. EphA4 protein levels were similar between the two groups. Student’s t-test, *n* = 6 mice per group, *P* > 0.05. (**C**) No differences in Epha4 and ephrin ligand expression in ipsilesional cortical brain tissue from mice in enriched versus standard housing conditions at day 36 after experimental stroke. Two-way ANOVA, *n* = 6 per group, *P* > 0.05. All data are presented as mean ± SD. GAPDH = glyceraldehyde 3-phosphate dehydrogenase, EE = enriched environment.

To evaluate potential differences in EphA4 and ephrin levels after changing environmental conditions, we determined EphA4 and ephrin ligand expression in the ipsi- and contralesional cortex at 5 weeks after experimental stroke. Exposure to an enriched environment did not change EphA4 protein (Fig. 5A and B) and mRNA levels (Fig. 5C) nor the expression of its ligands at day 36 of the experiment (Fig. 5C).

### Subacute EphA4 inhibition does not affect functional recovery after experimental stroke 

**Figure 6:**
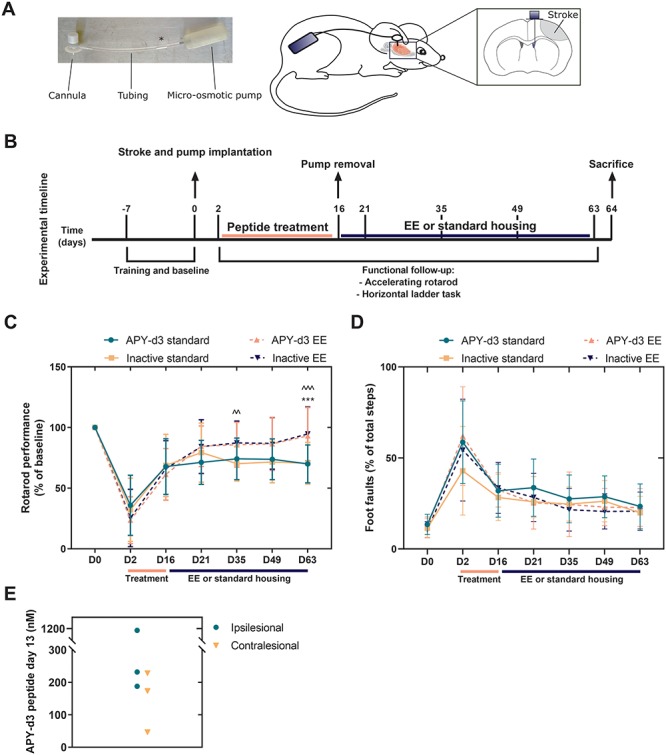
Environmental enrichment improves functional outcome after experimental stroke without additive beneficial effects by EphA4 targeted therapy. (**A**) Illustration of delayed APY-d3 peptide delivery using micro-osmotic pumps and ICV cannulas. Left image shows introduction of small air bubble (^*^) in catheter to separate aCSF from peptide solutions resulting in 48 h delayed treatment. Right illustration indicates pump implantation at the non-affected flank of the animal with ICV cannula placed in the ipsilesional lateral ventricle. (**B**) Experimental timeline. Mice were trained for 1 week on the accelerating rotarod and horizontal ladder task. After baseline assessments, cortical stroke was induced followed by implantation of micro-osmotic pumps and ICV cannulas during the same surgical procedure. aCSF was delivered for 48 h, followed by APY-d3 or inactive peptide treatment for 2 weeks. At day 16, post-stroke mice were functionally assessed and pumps removed. Mice were assigned to enriched or standard housing until day 63 post-stroke. (**C**) Functional motor recovery as measured with the accelerating rotarod. APY-d3 treatment did not influence motor recovery. Environmental enrichment started at day 16 and significantly increases functional improvements from Day 35 onwards. Two-way ANOVA, *n* = 16–24 mice per group, ^^*P* < 0.01 inactive standard versus inactive EE, ^^^*P* < 0.001 inactive standard versus inactive EE, ^***^P < 0.001 APY-d3 standard versus APY-d3 EE. (**D**) Functional motor recovery measured with the horizontal ladder task is not different between any of the groups tested. Two-way ANOVA, *n* = 16–24 mice per group, *P* > 0.05. (**E**) APY-d3 peptide detection in the ipsi- (mean = 530 nM) and contralesional (mean = 150 nM) hemisphere after 13 days ICV infusion. *n* = 3 mice per condition. All data are presented as mean ± SD. EE = enriched environment.

In order to assess the translational potential of rehabilitation with EphA4 targeted therapy, we implanted micro-osmotic pumps and ipsilesional ICV infusion cannulas during the same surgical procedure as the induction of stroke in C57BL/6J mice. A small air bubble at the beginning of the catheter separated aCSF-filled tubing from treatment solution, containing the EphA4 inhibitory or inactive peptide APY-d3 (30), allowing the 2-week treatment to start at 48 h after stroke (Fig. 6A). From day 2 after experimental stroke, functionally impaired mice received peptide treatment without behavioral assessments during the two treatment weeks. We removed micro-osmotic pumps at day 16 and randomly assigned mice to enriched or standard housing for the remaining 7 weeks of the experiment (Fig. 6B).

All animals showed functional deficits at day 2 post-stroke as shown by the time on the rotarod and the percentage of foot-faults on the horizontal ladder task (Fig 6C and D). After 2 weeks of treatment, mice receiving APY-d3 peptide treatment improved to similar levels as mice treated with inactive peptide (Fig. 6C and D). Exposure to an enriched environment resulted in a persistent functional improvement until the final follow-up at day 63 after stroke, without synergistic effects of EphA4 targeted therapy (Fig. 6C). Mice in the enriched environment almost completely recovered on the accelerating rotarod while mice in standard housing recovered to about 70% of baseline performance (Fig. 6C). In the horizontal ladder task, animals in the enriched environment recovered to the same extent as mice in standard housing conditions (Fig. 6D). In order to confirm proper peptide delivery, we assessed peptide concentrations in the brain at day 13 after pump implantation in a separate cohort of stroked C57BL/6J mice. APY-d3 concentrations were in the 150–200 nm range in ipsi- and contralesional brain tissue (Fig. 6E).

## Discussion

Recent studies indicate that axon growth-promoting therapies combined with rehabilitation might serve as optimal strategies to improve stroke outcome (23). In the present study, we show that rehabilitation during the chronic phase after photothrombotic stroke persistently improves functional outcome at least up to 2 months after injury without synergistic effects of EphA4 inhibition.

Subacute immunotherapy against one of the post-stroke growth-inhibitory proteins, Nogo-A, followed by intense rehabilitation improves recovery after large cortical stroke in rats (24). Here, animals almost fully regained forelimb motor function when treated with anti-Nogo-A antibodies during the first 2 weeks followed by intense training. Simultaneous treatment and rehabilitation during the subacute phase did not result in a beneficial outcome. To further evaluate the relevance of combined growth-promoting treatments and rehabilitation, we focused on EphA4, an inhibitor of axonal outgrowth. EphA4 might be an important regulator of stroke recovery since aged animals increase *Epha4* expression in post-stroke sprouting neurons compared to young animals (26). In addition, we showed that EphA4 reduction improves functional outcome after experimental stroke (25). In the present study, we first identified *Epha4* expression patterns after photothrombotic stroke. The majority of surviving neurons in the ipsilesional cortex expressed *Epha4* during the subacute and chronic phase after stroke. This expression pattern is in accordance with data showing *Epha4* expression in cortical, hippocampal and spinal cord neurons (31). Although astrocytic EphA4 influences glial scar formation and axonal outgrowth after spinal cord injury (32), we detected only limited *Epha4* expression in ipsilesional astrocytes at day 7 post-stroke. *Epha4* expression levels analyzed by the number of *Epha4* puncta/μm^2^ in neurons or astrocytes did not significantly change although a trend towards increased numbers of puncta in the stroke versus non-stroke condition was present for both cell types. Indeed, total cortical *Epha4* mRNA levels at the same time point post-stroke (day 7) were elevated. Additionally, EphA4 protein levels were increased from 24 h to 21 days after stroke. Although total EphA2/3/4/5 phosphorylation increases during the first week after middle cerebral artery occlusion (MCAO) (27), EphA4 phosphorylation relative to total EphA4 protein levels remained unaltered during the acute, subacute and chronic phase after photothrombotic stroke. Discrepancies between studies might be the results of model specific responses, differences between spatial tissue collection and/or detection of EphA4-specific versus general phosphorylation of multiple EphA receptors.

In rodent stroke models, different rehabilitation strategies are used. The introduction of an enriched environment is an easy *in vivo* procedure that can be translated to current clinical care for stroke patients (8). Starting environmental enrichment in the early subacute phase improves motor performance in mice after photothrombotic stroke (33). Additionally, stroke recovery in rats improves after environmental enrichment during both the early and late subacute phase (11). We designed a basic enriched environment for mice and show that this improves functional outcome after experimental stroke, even when started in the chronic phase. The environmental enrichment paradigm increased performance on the accelerating rotarod, while the use of the affected forelimb on the horizontal ladder task did not differ between mice in enriched versus standard housing. This suggests that the beneficial effect of environmental enrichment may partially involve compensatory behavioral adjustments as rotarod performance depends on body posture, balance, coordination and motor function, while the horizontal ladder task specifically detects changes in limb use (34,35). These results highlight the importance of increased social and sensorimotor stimuli in post-stroke functional performance. In contrast to changes in Eph receptor and ephrin expression after skilled reaching tasks (36), changing environmental conditions did not modify *Epha4* and ephrin ligand expression.

EphA4 inhibition by APY-d3 peptide treatment for 2 weeks starting at day 2 after experimental stroke did not result in beneficial effects on stroke recovery. Mice treated with either APY-d3 or control peptide showed similar motor performance at the end of the treatment. Additionally, EphA4 targeted therapy did not result in synergistic beneficial effects on functional outcome when rehabilitation was initiated after pharmacological treatment. Both APY-d3 and control peptide-treated mice showed improved motor performance upon environmental enrichment with no difference between the treatment groups. Using ephrin-A5 ligand binding ELISAs, we detected 150–200 nm peptide concentrations in the ipsi- and contralesional hemisphere after 13 days of infusion. These concentrations are sufficient to completely inhibit growth cone collapse in retinal explants (30). However, in HEK293 cells, they only inhibit EphA4 phosphorylation by 30–40% when stimulated with ephrin-A5 (30). Thus, it is possible that higher levels of EphA4 inhibition are needed in order to achieve benefits on stroke outcome. In addition, the APY-d3 peptide targets the EphA4 receptor with high specificity (30), and therefore it only inhibits EphA4 signaling without affecting other Eph receptors. Since additional Eph receptors may participate in post-stroke plasticity and recovery (27), widespread Eph-ephrin signaling inhibition might be required to enhance post-stroke plasticity and restore motor function (25,27). For this purpose, the combination of rehabilitation and targeting Eph signaling with a pan Eph antagonist like EphA4 Fc, UniPR1331 or GLPC1790 might be of interest (27,37–39).

Our study has several limitations. We measured peptide levels in ipsi- and contralesional brain tissue in a subset of mice showing abundant APY-d3 levels in both hemispheres. However, peptide concentrations were variable between animals. This variability might be due to the release of some concentrated peptide solution upon removal of the cannula after sacrificing the animals, especially since only one sample showed such a high peptide concentration. Unfortunately, we were not able to directly link peptide concentration to the extent of recovery because mice were continuously followed-up after peptide infusion during the enriched versus standard housing experiment until day 63 after experimental stroke. We used the accelerating rotarod and horizontal ladder task to assess functional improvements after stroke. Environmental enrichment increased rotarod performance, while the number of foot faults on the horizontal ladder remained unaltered. These results suggest possible involvement of compensatory movements. To distinguish between restorative and compensatory mechanisms, additional behavioral tests, for example skilled reaching paradigms (40), would have been of interest. Furthermore, we did not assess the effect of the enriched environment and/or EphA4 targeted therapy on mechanisms underlying stroke recovery, including axonal plasticity and glial scar formation. Large cortical lesions affecting corticospinal projections induce sprouting from the intact contralesional corticospinal tract (CST) into the denervated cervical spinal cord (41), and post-stroke rehabilitative training enhances CST re-innervation leading to improved functional outcome (42). Since EphA4 regulates CST formation during development and EphA4 inhibition results in increased axonal regeneration after spinal cord injury (43), it would be interesting to identify CST re-innervation in the denervated cervical spinal cord upon different treatment and rehabilitation paradigms. Additionally, EphA4 affects astrocyte reactivity after spinal cord injury resulting in enhanced axonal plasticity after EphA4 inhibition (32). Because reactive astrocytes and post-stroke plasticity are closely linked (17), the contribution of environmental enrichment and EphA4 inhibition on astrocytic phenotypes, glial scar formation and axonal plasticity would be an interesting mechanism to assess. However, since we did not observe any synergistic effects of EphA4 targeted therapy, we concluded that these experiments lack relevance for further translational implications. We therefore did not pursue these strategies.

Overall, our findings indicate that an enriched environment improves functional outcome even when initiated in the chronic phase after experimental stroke and that the subacute EphA4 targeted therapy we employed does not have additional beneficial effects.

## Materials and Methods 

### Animals, housing and study approval 

In-bred C57BL/6J mice (C57BL/6Jax stock 000664) were originally purchased from The Jackson Laboratory (Ben Harbor, ME, USA). EphA4^LacZ^ mice on a C57BL/6J background were kindly provided by Professor R. Klein (Max-Planck-Institute of Neurology, Munich, Germany). In these mice, the endogenous EphA4 promotor is followed by a LacZ and a human placental alkaline phosphatase (PLAP) gene, producing a EphA4/β-galactosidase (β-gal) fusion and a PLAP protein (28).

Mice were maintained under specific pathogen-free conditions at the KU Leuven animal facility under a 12-h light/dark cycle with ad libitum access to standard rodent chow and water. The room temperature was maintained at 20°C. Mice were transferred to experimental rooms 1 week before training. Adult (10–12 weeks of age), male mice were included in the study. Weight was carefully monitored and did not drop during the course of the experiment. All experiments were in accordance with the Guide of Care and Use of Experimental Animals of the Ethical Committee of KU Leuven and followed the ARRIVE guidelines. The Ethical Committee of KU Leuven approved all animal experiments (P015/2014 and P132/2018).

### Stroke model 

Focal ischemia in the forelimb motor cortex was induced using the photothrombotic lesion model (44,45) resulting in infarcts covering 20% of the hemisphere (46). Briefly, mice were anesthetized with 2.5% isoflurane (halocarbon) in an oxygen/air mixture, respiration was monitored and rectal temperature was maintained at 37 ± 0.5°C using a heating plate (TCAT-2LV Controller, Physitemp Instruments, Clifton, NJ, USA). After fixation in a stereotactic frame (David Kopf Instruments, Tujunga, CA, USA), the skull was exposed by a 1 cm midline incision of the skin. An amount of 100 μl Rose Bengal (Sigma) at a concentration of 3 mg/ml saline was infused by tail vein injection. For illumination, a 2.4 mm laser beam with a wavelength of 565 nm (L4887-13, Hamamatsu Photonics, Japan) was focused on the motor cortex responsible for forelimb function (0.5 mm rostral, 1.8 mm lateral of bregma). Five seconds after Rose Bengal injection, the brain was illuminated though the intact skull for a total duration of 5 min. After illumination, the incision was sutured and 500 μl saline and 0.05 mg/kg Vetergesic (Ecuphar) was given to the animals subcutaneously. To recover, mice were placed in a separate cage with half of the cage placed under an ultraviolet lamp before returning to their home cage and housing facility. After stroke, mice were monitored on a daily basis during the first week and from then on animal health was checked weekly. To prevent unnecessary suffering of animals, mice were euthanized if they showed severe weakness or if they lost 20% of their body weight within 5 days.

### Intracerebral ventricular peptide treatment 

One day prior to surgery, micro-osmotic pumps were prepared according to the manufacturer’s protocol (Alzet, Cupertino, CA, USA). Briefly, micro-osmotic pumps with a flow rate of 0.25 μl/h (Pump model 1002, Alzet) were probed with peptide solution; 5 mm APY-d3 (βAPYCVYR βASWSC) (30) or 5 mm inactive peptide (βAPYCVYRβASSWC) (Eurogentic, Liege, Belgium) in fresh artificial cerebrospinal fluid (aCSF, Alzet). After 6 h probing, brain intracerebral ventricular (ICV) infusion cannula’s (Brain infusion kit 3, Alzet) were attached by a 3.6 cm catheter. All catheters were filled with aCSF and a small air bubble on the site of the pump, separating treatment solution from the aCSF filled catheter. A catheter length of 3.6 cm was used to ensure aCSF treatment within the first 48 h after pump placement for all treatment groups. Pumps were incubated at 37°C before implantation.

Photothrombotic lesions were induced as described above but with the following adjustments. After exposing the skull, a small hole was made using a 28 gage drill (Bilaney, Düsseldorf, Germany) attached to the stereotactic frame at the position of the ipsilesional lateral ventricle (0.1 mm caudal and 1.0 mm lateral of bregma). After lesion induction, cannulas were positioned and mounted to the skull using Loctite 454 adhesive glue (Loctite, Rocky Hill, CT, USA). Cannula tabs were removed. Micro-osmotic pumps were placed subcutaneously on the non-affected side of the animal’s back, the wound was sutured and animals were given 500 μl saline and 0.05 mg/kg Vetergesic (Ecuphar) subcutaneously. To exclude any neuroprotective effects of the peptide treatment, all animals received aCSF within the first 48 h after experimental stroke. At day 16 post-stroke, micro-osmotic pumps were removed via a small incision. Remaining catheters were closed using a small vessel cauterizer (Fine Scientific Tools, Heidelberg, Germany). After pump removal mice were housed under standard or enriched housing conditions (see below).

### Peptide detection

To measure peptide concentrations after infusion, three stroked mice were infused with 5 mm APY-d3 and three stroked mice were infused with 5 mm inactive peptide. Mice were sacrificed by cervical dislocation at day 13 after pump implantation. Brains were dissected, frontal lobes and cerebellum were removed and the ipsi- and contralesional hemisphere were collected. Tissue was snap-frozen in liquid nitrogen and stored at −80°C. Peptide concentration in brain lysates was measured using a previously described ligand-binding ELISAs (30). Briefly, brain tissue was homogenized in ice-cold TBST (150 mm NaCl, 50 mm Tris HCl pH 7.5, containing 0.01% Tween-20) using a polytron (PT1600 E, Kinematica, Bohemia, US). Homogenized tissue was incubated on a tube rotator for 1 h at 4°C and centrifuged at 16 000*g* for 10 min at 4°C. The supernatant, containing the peptide, was collected and used to measure inhibition of the binding of ephrin-A5 fused to alkaline phosphatase (ephrin-A5 AP) to EphA4 Fc immobilized in the wells of a 96 well plate. EphA4 Fc (Novoprotein) was immobilized at 1 μg/ml on protein A coated 96-well plates (Pierce-Thermo Scientific) for 1 h at room temperature in TBST. The plates were washed three times with TBST and incubated for 1 h at 4°C with 0.05 nm ephrin-A5 AP and eight different dilutions of the homogenized brain supernatant (obtained by 2-fold serial dilution starting from the non-diluted supernatant). After washing away unbound ephrin-A5 AP, the amount of bound ephrin-A5 AP was quantified by using p-nitrophenyl phosphate substrate (Pierce-Thermo Scientific) diluted in SEAP buffer (105 mm diethanolamine, 0.5 mm MgCl_2_, pH 9.8). After 1 h incubation at 37°C, optical density at 405 nm (OD_405_) was measured. OD_405_ values from wells coated with Fc alone were subtracted as the background and OD_405_ values from brain supernatant containing inactive peptide were used to determine maximal ephrin-A5 AP binding. Peptide concentrations in brain lysates were calculated based on a standard inhibition curve generated with known concentrations of APY-d3 peptide. Brain concentrations were calculated by taking into account the amount of lysis buffer added to brain tissue (2:1 by weight).

### Enriched environment 

To test the effectiveness of environmental enrichment on stroke recovery, mice were housed in standard cages (370 cm^2^) or in enriched cages with increased social, sensorimotor and cognitive stimulations. Eight to ten animals were placed into a larger cage (800 cm^2^) with different rodent toys (from day 2 onwards). Toys included a climbing ladder, running-wheel, chains, different-shaped objects and plastic tunnels. Standard housing included 3–5 animals per cage without additional toys. To test the efficacy of the enriched environment as rehabilitation, mice were functionally assessed for 5 weeks after stroke using the accelerating rotarod. In addition, non-stroke animals were housed under the same conditions to determine the effect on general activity and motivation to perform functional tests.

To determine a potential synergistic beneficial effect of the enriched environment with EphA4 targeting peptide treatment, the same housing conditions were applied to animals that received 2-week APY-d3 or inactive peptide treatment. After pump removal at day 16 after stroke, mice were assigned to standard or enriched housing conditions until the end of the experiment (day 63).

### Behavioral testing 

To assess behavioral improvements after experimental stroke, the accelerating rotarod and horizontal ladder task were used. The horizontal ladder set-up were developed in collaboration with the mechanical maintenance facility at the KU Leuven and was composed of two Plexiglas walls containing holes to create irregular bar patterns (35). Mice were trained for 1 week before stroke and functional assessments were done at day 2 to ensure clear motor defects. Behavioral outcome was determined by weekly or two-weekly assessments for the rest of the experiment. All tests were performed around the same time during the day by an investigator blinded to the treatment. Mice were placed in the test room 30 min before testing to acclimatize.

*Accelerating rotarod.* To examine motor function, balance and coordination, the accelerating rotarod (34,47) was used. This test involves a rotating cylinder with a rotational speed of 4–40 rpm over a period of 300 s. During test sessions, each mouse was given three attempts to walk on the treadmill and latency in time for the animals to fall off the cylinder was recorded. The average time on the treadmill was used as the final score per animal. Prior to experimental stroke, mice were trained for 1 week followed by a baseline assessment that was used to normalize test results after photothrombotic stroke to correct for individual variation. Animals that performed above 75% of baseline performance at day 2 post-stroke were excluded for further follow-up.

*Horizontal ladder task:* The horizontal ladder task (35,48) was used to measure the animals’ ability to properly place forelimbs on a non-equidistant grid (space between bars was between 0.5 and 2.5 cm). The ladder was composed of two Plexiglas walls (69.5 × 15 cm) spaced 5 cm apart. Mice were placed on one side of the ladder and had to walk towards their home cage on the other side of the ladder. Each animal crossed the ladder three times during a session and after each session bars were changed to prevent animals from memorizing the bar pattern. Using a video recording, each step was scored based on the foot placement. A step was considered a foot fault when the paw did not contact the bar, slipped through it or was placed incorrectly. Foot faults as a percentage of the total number of steps over three crossings was used as the final score per animal. Mice that were excluded based on rotarod performance were not included in this task.

### RNA *in situ* hybridization 

Non-stroke and stroked C57BL/6J mice were anesthetized with an overdose of Dolethal (20 mg/ml, Vetoquinol) and transcardially perfused with RNAse-free PBS (Sigma-Aldrich) followed by 4% PFA (Life Technologies). Brain tissue was post-fixed with 4% PFA overnight, followed by cryoprotection in 30% sucrose. Tissue was snap-frozen and embedded in OCT embedding matrix (CellPath). 40 μm cryosections were obtained with a CryoStart NX70 Cryostat (ThermoFisher Scientific) and mounted on Superfrost Plus slides (ThermoFisher Scientific). On the first day, sections were dried at room temperature and a tissue barrier was created using a hydrophobic pen (Immedge, Vector Labs). Post-fixation was done for 30 min at 4°C in ice-cold 4% PFA after which glasses were washed three times in 1× RNAse free PBS followed by dehydration for 5 min in 50% ethanol (Sigma-Aldrich), 5 min in 70% ethanol and 5 min 100% ethanol at room temperature. Sections were baked for 30 min at 60°C and the RNAscope *in situ* hybridization protocol was performed using the commercially available RNAscope kit (Advanced Cell Diagnostics) using the following probes: Mm*-Epha4* C1 (ref 419 081), Mm-*Slc1a3* C2 (ref 430 781), Mm-*Syp* C3 (ref 4 265 213), 3-Plex Negative Control Probe (ref 320 871). In brief, slides underwent an antigen retrieval step of 5 min at 98–104°C with a Braun Multiquick FS-3000 Steamer (Braun) and a Protease III incubation step of 30 min at 40°C in a HybEZ™ Oven (ACD Diagnostics, Bio-Techne, Abingdon, UK). Sections were incubated for 2 h at 40°C in the HybEZ™ Oven with the appropriate RNAscope probes. Signal amplification and detection was done with the RNAscope multiplex fluorescent reagent kit V2 combined with the TSA Plus Cyanine 3 system as stated in the manufacturer’s instructions (NEL7600001, Perkin Elmer). Cyanine 3 (1:500) was used to detect *Epha4*, cyanine 2 (1:2000) for *Syp* and cyanine 5 (1:2000) for *Slc1a3*. For nuclei detection, Hoechst 33342 staining (Sigma-Aldrich, 1:2500) was performed. Slides were mounted with ProLong Gold antifade reagent (Life Technologies, Carlsbad, USA). Within the ipsilesional motor cortex responsible for the use of the forelimb, two pictures per animal were taken with a Leica TCS SP8 confocal microscope with an HC PL APO CS2 20X/0.75 dry lens and a pinhole of 0.5 AU (Leica Microsystems Heidelberg GmbH, Manheim, Germany). Projections of four Z-stacks separated 2 μm from each other was done with Fiji, and images were automatically quantified with the NIS-Elements Microscope Imaging Software (Nikon). Analysis involved nuclei detection, followed by the intensity of *Syp* (neuronal maker), presence of *Slc1a3* (astrocyte marker) and number *Epha4* puncta. The number of *Epha4* puncta in cells positive for either *Syp* or *Slc1a3* was counted, and the total number of *Epha4+* cells as a percentage of the total *Syp+* and *Slc1a3+* cells was calculated. A cell was considered positive if it contained at least two *Epha4* puncta. In order to measure the extent of expression, the average number of puncta per μm^2^ in *Syp +* neurons and *Slc1a3+* astrocytes was assessed.

### Western blot 

Mice were sacrificed at various time points after PTL by cervical dislocation. Brains were dissected and positioned in a mouse brain matrix (Zivic Instruments, Pittsburgh, USA), and a 5 mm thick section around the lesion was isolated. Cortical tissue surrounding the infarct and the corresponding area in the contralesional hemisphere was collected using a biopsy plunger. Tissue was snap-frozen in liquid nitrogen and stored at −80°C.

For western blot analysis, cortical tissue was homogenized in RIPA buffer (Sigma-Aldrich) supplemented with Complete EDTA-free Cocktail (Roche), phosSTOP (Roche) and PMSF (ThermoFischer Scientific), by means of mechanical disruption using Lysing Matrix D beads (MP Biomedicals, Santa Ana, CA, USA) and a MagNa Lyser oscillator (Roche, Basel, Switzerland) three times at 6500 rpm for 30 s with 1 min interval on ice. Homogenized tissue was incubated on ice for 30 min and centrifuged at 13 000 rpm for 10 min at 4°C. Supernatant was collected as total protein samples. Protein concentration was determined using a BCA kit (Thermofisher Scientific) and 15 μg of protein lysate with Pierce Lane Marker Reducing Sample Buffer (ThermoFischer Scientific) was heated for 5 min at 95°C. Protein was loaded on a polyacrylamide gel, subjected to gel electrophoresis and transferred onto a polyvinylidene difluoride (PVDF) membrane (Millipore). Membranes were blocked for 1 h at room temperature with 5% non-fat milk (Sigma-Aldrich) and incubated overnight at 4°C with the appropriate antibody; mouse-anti-EphA4 (Invitrogen, 37-1600, 1:500). The next day, membranes were incubated with peroxidase-labeled secondary antibody (DAKO, 1:5000). Immunoreactivity was visualized using enhanced chemiluminescence (ECL, Thermofisher Scientific) and an ImageQuant LAS4000 imager (GE Healthcare, Machelen, Belgium). α-Tubulin (Sigma, T6199, 1:5000) or GAPDH (Ambion, ThermoFisher Scientific, 1:10 000) were used as reference proteins. ImageQuant LAS4000 software (GE Healthcare) was used for analysis.

### Immunoprecipitation 

From cortical extracted protein lysate, 500 μg was immunoprecipitated using Dynabeads protein G immunoprecipitation kit (Thermofisher Scientific), with 5 μg EphA4 antibody (Life Technologies) per sample, and 5 μg anti mouse IgG1 (R&D) was used as isotype control. Immunoprecipitates were subjected to western blotting as described above. To detect EphA4 phosphorylation, membranes were probed with rabbit-anti-EphA4(Tyr602) antibody (ECM Biosciences, 1:500) followed by stripping and reprobing with mouse-anti-EphA4 antibody (Life Technologies, 1:500). Blots were visualized and analyzed as described above and EphA4 phosphorylation was calculated as the percentage of total EphA4 levels.

### RNA isolation and qPCR 

Mice were sacrificed by cervical dislocation and cortical tissue was collected as described above (western blot paragraph). Total RNA was extracted using Tripure (Roche) and isopropanol (Sigma-Aldrich) purification. cDNA was synthesized using the SuperscriptTM III First-Strand Synthesis Mastermix kit (Thermofisher Scientific) and quantitative PCR reactions were performed using 5 μl cDNA and predesigned qPCR assays (IDT). Thermal cycling was done on a StepOne-Plus Real-Time PCR system (Applied Biosystems, Foster City, USA) using a standard amplification protocol with Taqman assays (IDT). Data were processed and analyzed in qBase Plus software (Biogazelle, Gent, Belgium). The following 20× assays were used: Epha4 (Mm.PT.58.13545379), Efna1 (Mm.PT.58.29850712), Efna2 (Mm.PT.58.11885886), Efna3 (Mm.PT.58.10380528), Efna4 (Mm.PT.58.9770779), Efna5 (Mm.PT.58.28681125), Efnb1 (Mm.PT.58.28819484), Efnb2 (Mm.PT.58.29108694), Efnb3 (Mm.PT.58.41654515), Polr2a (Mm.PT.58.13811327), Ywhaz (Mm.PT.39a.22214831) and Gapdh (Mm.PT.39a.1).

### Immunostainings 

Mice were anesthetized with Dolethal (20 mg/ml) in PBS and transcardially perfused with PBS followed by 4% PFA. Brains were post-fixed in 4% PFA at 4°C overnight, followed by dehydration in 10, 20 and 30% sucrose overnight (Sigma-Aldrich). Tissue was snap-frozen in ice-cold isopentane (Sigma-Aldrich) and 40 μm coronal-free floating cryosections were collected in 0.02% sodium azide (Sigma-Aldrich) in PBS in 96-well plates (Sigma-Aldrich) using a CryoStart NX70 Cyrostat. Sections were blocked for 1 h at room temperature in 10% normal donkey serum (Sigma-Aldrich) and stained overnight at 4°C using antibodies recognizing β-galactosidase (Abcam, 9361, 1:2000), CD11b (AcD Serotec, MCA74G, 1:200), NeuN (Millipore, MAB377, 1:200) and GFAP (Sigma, G3893, 1:500). After incubation with the appropriate secondary antibody conjugated to an Alexa-fluophore (Life Technologies), nuclei were visualized using Hoechst 33342 counterstain (1:750) and sections were mounted with Prolong Gold antifade mounting agent (Thermofisher Scientific). Three Z-stack confocal images (four stacks of 2 μm apart) per animal were taken within the perilesional motor cortex responsible for the use of the forelimb using a Leica TCS SP8 confocal microscope (Leica) with a 20×, 40× or 63× objective. Maximum projections of Z-stacks were made and analyzed using Fiji (ImageJ).

### Statistical analysis 

A priori power analysis based on previous results (25,27) to detect a relevant 17% difference on behavioral analysis with 80% power (*α* = 0.05) estimated a sample size of 26 animals per group. All data were analyzed using GraphPad Prism 8 software (Graphad Software). Specific statistical tests are described in figure legends. Non-parametric testing was performed when data were not normally distributed (Shapiro-Wilk normality test). ^*^P < 0.05 was considered as statistically significant. Smaller *P*-values were represented as ^**^P < 0.01, ^***^P < 0.001 and ^****^P < 0.0001. Data are represented as mean ± SD.
